# Diagnostic Accuracy of Ultrasound for Identifying Shoulder Dislocations and Reductions: A Systematic Review of the Literature

**DOI:** 10.5811/westjem.2017.5.34432

**Published:** 2017-07-10

**Authors:** Michael Gottlieb, Frances Russell

**Affiliations:** * Rush University Medical Center, Department of Emergency Medicine, Chicago, Illinois; † Indiana University School of Medicine, Department of Emergency Medicine, Indianapolis, Indiana

## Abstract

**Introduction:**

Patients with shoulder dislocations commonly present to the emergency department. Ultrasound has the potential to save time, radiation exposure, healthcare costs, and possible need for re-sedation. We conducted this systematic review to compare the diagnostic accuracy of ultrasound compared with plain radiography in the assessment of shoulder dislocations.

**Methods:**

We searched PubMed, Scopus, the Cochrane Database of Systematic Reviews, and the Cochrane Central Register of Controlled Trials for relevant trials. Primary data and test characteristics were obtained for all included studies. We used QUADAS-2 to assess study quality. Meta-analysis was not performed due to significant heterogeneity.

**Results:**

Four studies met our inclusion criteria, comprising 531 assessments with 202 dislocations. Most studies had a sensitivity of 100% for identifying dislocations. One study demonstrated a sensitivity of 54%, and another had only one dislocation that was misidentified. All studies were 100% specific for detecting dislocation.

**Conclusion:**

Ultrasound may be considered as an alternative diagnostic method for the detection of shoulder dislocation and reduction, but further studies are necessary before routine use.

## INTRODUCTION

Shoulder dislocations are a common presentation to the emergency department (ED) with an estimated incidence of 23.9 dislocations per 100,000 person-years in the United States.^1^ These injuries affect 1.7% of the population, resulting in 200,000 ED visits each year.^2^ Most shoulder dislocations are reduced in the ED with radiographs performed to both identify the dislocation and confirm the reduction. With increasing availability and comfort with ultrasound (US), multiple case reports have suggested that US may be a valuable adjunct for identifying dislocations and confirming reductions.^3^–^6^ Using US for the assessment of shoulder dislocations and reductions may save time, radiation exposure, healthcare costs, and the potential need for re-sedation in select patients (due to more rapid identification of unsuccessful reductions). However, it is important to ensure that this technique is accurate and reliable before routine clinical application.

We conducted a systematic review to determine the diagnostic accuracy of US to detect shoulder dislocation and reduction when compared with plain radiographs.

## METHODS

We conducted a systematic search of PubMed, Scopus, the Cochrane Database of Systematic Reviews, and the Cochrane Central Register of Controlled Trials to include citations from inception to April 3, 2017, using a combination of the keywords and Medical Subject Headings (MeSH) “shoulder dislocation,” “shoulder relocation,” “shoulder reduction,” and “ultraso*” with no limitations or language restrictions (Appendix). We reviewed the bibliographies of identified studies and review articles for potential missed articles. We also consulted with topic experts to help identify any further relevant studies.

Inclusion criteria included all original, published, primary research articles assessing the accuracy of US for identifying shoulder dislocation and/or reduction. We included prospective, observational studies and randomized, controlled trials. Review articles, case reports, case series, retrospective reviews, and isolated abstracts were excluded. Two physician-investigators independently assessed studies for eligibility based upon the above criteria. All abstracts meeting initial criteria were reviewed as full manuscripts. Studies determined to meet the eligibility criteria on full text review by both extractors were included in the final data analysis. Any discrepancies were resolved by consensus.

Two physician-investigators independently extracted data from the included studies into a data collection form. The following information was abstracted: last name of the first author, study title, publication year, study design, total study population size, total number of dislocations within the study population, US machine, US probe type, US training protocol, US criteria for the diagnosis of shoulder dislocation, gold standard for the diagnosis, true positives, true negatives, false positives, and false negatives. We assessed studies for quality using the Quality Assessment of Diagnostic Accuracy Studies (QUADAS-2) tool.^7^

We created two-by-two contingency tables for each study with sensitivities and specificities and 95% confidence intervals derived from this data. To standardize the interpretation, we determined sensitivity and specificity with respect to the identification of shoulder dislocation regardless of whether the exam was performed before or after a reduction attempt. Data were not combined for meta-analysis due to significant clinical heterogeneity with regard to training and US protocol.

## RESULTS

The search of PubMed yielded 154 total studies. Scopus identified 243 total studies. Cochrane Central Register of Controlled Trials located six studies and the Cochrane Database of Systematic Reviews identified no further studies. Of the 403 total studies identified with this search strategy, only four met the inclusion criteria and were included in this review (Figure).

All four studies were prospective, observational studies comparing US with conventional radiography for assessing shoulder dislocations and/or reductions. Two studies included data sets for both initial dislocation assessment and subsequent relocation assessment separately.^8^,^9^ There were a total of 531 total assessments performed with 202 shoulder dislocations (38%); 260 (49%) were performed to assess for the initial dislocation and 271(51%) assessments were performed to assess for persistent dislocation after the initial reduction attempt. All assessments were performed in an ED setting.

Population Health Research Capsule

What do we already know about this issue?

Shoulder dislocations are a common presentation to the emergency department. Ultrasound has been proposed as an alternate diagnostic modality in place of radiographs.

What was the research question?

This systematic review was performed to determine the diagnostic accuracy of ultrasound for identifying shoulder dislocations.

What was the major finding of the study?

Ultrasound was both sensitive and specific for identifying shoulder dislocations, but further studies are needed.

How does this improve population health?

If supported with additional data, ultrasound may be used in place of radiography to save time, radiation exposure, healthcare costs, and the potential need for re-sedation.

The studies varied with respect to the US training protocol, ranging from reliance on existing experience^8^,^11^ to various combinations of lectures and hands-on practice.^8^–^11^ The US examinations also significantly varied between studies. Abbasi et al. used an anterior and lateral approach.^8^ The anterior technique involved visualizing the coracoid process and humeral head assessing for the position of the humeral head (i.e., inferior in dislocation and lateral in reduction). The lateral approach involved visualizing the acromion process and the humeral head assessing for the proximity of the humeral head (i.e., wide in dislocation and narrow in reduction). Aykol et al. traced the humerus from the posterior aspect to view the glenohumeral joint.^9^ Dislocation was suggested by an inferiorly displaced humerus, posteriorly displaced humerus, or lack of rotational articulation on internal and external rotation. Lahham et al. placed the transducer in a transverse orientation on the posterior aspect of the patient’s shoulder and measured the distance between the glenoid fossa and humeral head with a positive distance representing an anterior dislocation, a negative distance representing a posterior dislocation, and zero centimeters representing normal anatomic alignment.^10^ Ahmadi et al. visualized the glenoid fossa from both an anterior and lateral direction, though they did not further describe their protocol or measurements.^11^

Most studies were 100% sensitive, with two studies having less than 100% sensitivity (Table 1). Ahmadi et al. demonstrated 53.8% sensitivity in confirming persistent dislocation after a reduction attempt among 108 patients with 13 dislocations.^11^ Akyol misidentified the one persistent dislocation as reduced among 94 patients after a reduction attempt.^9^ Specificity was 100% in all studies. As discussed above, meta-analysis was not performed due to significant differences with respect to the protocols.

Using the QUADAS-2 tool, all studies were deemed at overall low risk of bias (Table 2). All four studies used convenience sampling, so there was unclear risk of bias with respect to patient selection. Ahmadi et al.^11^ had unclear risk of bias with respect to reference standard due to the use of a single-view radiograph for confirmation of joint reduction. Additionally, the attending emergency physician’s interpretation of the post-reduction radiograph, who was not blinded to the patient, served as the criterion standard. Aykol et al.^9^ had unclear applicability concerns for the index test due to the use of two different types of US transducers for the exam.

## DISCUSSION

This systematic review suggests that US is sensitive and highly specific for the diagnosis of shoulder dislocation. All studies assessing the accuracy of US for detecting shoulder dislocation and reduction identified shoulder dislocation with 100% specificity.^8^–^11^ Most studies were also 100% sensitive, with the exception of one study demonstrating a sensitivity of 54%^11^ and another demonstrating misidentification of the only dislocation.^9^ Of note, the study with a sensitivity of 54% suffered from a number of methodologic flaws including unclear sonographer training, unclear US protocol, and an inadequate criterion standard (i.e., single view antero-posterior shoulder radiograph).

There was one prior systematic review published on this topic in 2016.^12^ However, this review was performed prior to the publication of the three most recent studies^9^–^11^ and provides only a short review of the existing evidence. The current review expands upon this by performing an updated review and using multiple databases to identify all relevant studies.

The use of US to identify shoulder dislocations and reductions has the potential to save patients time. One study demonstrated that the pre-reduction radiographs alone increased time to treatment by 30 minutes.^13^ By using point-of-care ultrasound (POCUS), the provider may reduce the total time that the patient spends in the ED and improve throughput efficiency. The reduction in time to imaging may be particularly important for patients undergoing procedural sedation. Rather than waiting for the patient to recover and sending him or her to the radiology suite for confirmation, the use of POCUS could allow rapid identification of a persistent shoulder dislocation. This would allow repeat reduction while the patient remains sedated, rather than having to repeat the procedural sedation. While the isolated radiation associated with a single radiograph is low, patients with shoulder dislocations may undergo several series of radiographs during their initial presentation, as well as during repeat dislocations. The use of US could reduce their total radiation exposure significantly over time. Finally, the use of repeated radiographs increases costs to both the patient and healthcare system. Incorporating US could have significant healthcare cost implications, especially given the high incidence and prevalence of this condition.^1^,^2^

As with all US applications, there is potential operator variability depending upon US skills. However, in the three studies in which training was described,^8^–^10^ providers demonstrated excellent accuracy despite short training sessions, suggesting that shoulder sonography for dislocation and relocation may have a short learning curve.

The variation in examination protocols does pose a challenge. However, the high sensitivity and specificity for shoulder dislocation identification in these studies suggests that multiple different sonographic approaches may be used to make this diagnosis. Two studies used different variations on an anterior and lateral approach,^8^,^11^ while the other two studies used variations on a posterior approach.^9^,^10^ Interestingly, Lahham et al. was the only study to use a numerical cut-off value.^10^ Future studies should compare the different techniques to determine which technique is the most accurate with a focus on standardizing techniques.

## LIMITATIONS

While the overall data is favorable, it is important to consider several limitations to the above studies. First, each study used a different protocol to assess for shoulder dislocation and reduction, which limits the ability to combine the test characteristics. Additionally, there were significant variations in training, ranging from specialty training in shoulder sonography to inexperienced undergraduate researchers.^8^,^10^ While this does result in increased heterogeneity, it also suggests that the learning curve may not be as steep as with other US applications. Another limitation is the potential for physical examination findings to influence the sonographer’s interpretation. While this may bias the potential of US to diagnose dislocation in isolation, we believe this is acceptable because sonographers will always be exposed to physical examination findings when performing an US examination.

It is important to note that the majority of dislocations assessed were anterior with only two posterior dislocations identified, thereby limiting the ability to extrapolate to posterior dislocations.^8^ Furthermore, the small proportion of non-dislocated shoulders on initial assessment in most studies resulted in wider confidence intervals (CI) and a lower limit of the CI for specificity as low as 50%.^9^ While the overall data is quite favorable, it is possible that the true specificity may be lower than suggested and more investigation is needed to validate this data. Finally, as US is operator-dependent, it is important to ensure that providers have undergone sufficient training and are aware of their limitations.

## CONCLUSION

While the data is supportive of the use of ultrasound for the diagnosis of shoulder dislocation, further studies are needed prior to routine implementation. Future studies should compare the different techniques to determine which is most accurate, record performance time for the ultrasound, include more data on posterior dislocations, include more data on fracture identification, and validate one of the above techniques with increased sample sizes.

## Supplementary Information



## Figures and Tables

**Figure:**
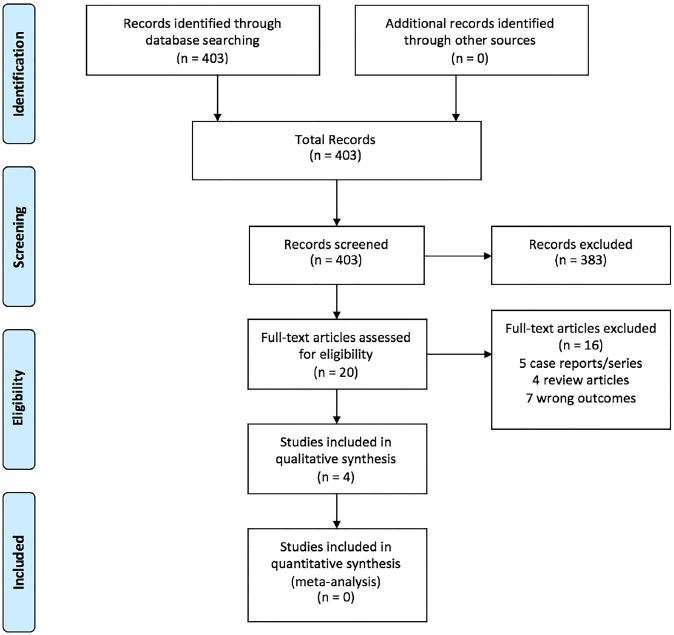
Outline of study selection and inclusion.

**Table 1:** Summary of existing studies on the accuracy of ultrasound for shoulder dislocation and reduction.

Study	Study design	Study population size (% dislocated)	Ultrasound probe and machine	Ultrasound training	Examination protocol	Sensitivity (95% CI)	Specificity (95% CI)
Abbasi 2013^8^	Prospective, observational	73 (94.5%)^A^	7.5–10 MHz linear transducer, SonoAce X8	Sonographer #1 prior experience (>5 years musculoskeletal ultrasound) Sonographer #2 1-hour lecture and 10 shoulder sonographic procedures supervised by the first sonographer	Anterior (coraco-humeral distance) and lateral (acromio-humeral distance) technique	100% ^A^ (93.4%–100%)	100% ^A^ (39.5%–100%)
		69 (2.9%)^B^				100%^B^ (19.7%-100%)	100%^B^ (93.2%–100%)
Akyol 2016^9^	Prospective, observational	103 (95.1%)^A^	7.5 MHz linear transducer, Mindray M5 and ESAOTE	30- minute lecture and two hours of hands-on practice	Posterior view of glenohumeral joint and assessment of rotational articulation on internal and external rotation	100% ^A^ (96.3%–100%)	100% ^A^ (47.8%–100%)
		94 (1.1%)^B^				0%^B^ (0%–97.5%)	100%^B^ (96.1%–100%)
Lahham 2016^10^	Prospective, observational	84 (22.6%)^A^	5–10 MHz linear transducer, Sonosite Edge	30-minute lecture and 30 minutes of hands-on practice	Single view measurement of glenohumeral separation distance	100%^A^ (82.4%–100%)	100% ^A^ (94.5%–100%)
Ahmadi 2016^11^	Prospective, observational	108 (12.0%)^B^	7 MHz linear transducer, Honda	Ultrasound training course in the radiology department	Anterior and lateral views of the humerus and glenoid fossa	53.8%^B^ (29.1%–76.8%)	100%^B^ (96.1%–100%)

*CI*, confidence interval; *A*, assessment of initial dislocation; *B*, assessment of persistent dislocation after reduction attempt.

**Table 2:** QUADAS-2 assessment.

	Risk of bias				Applicability concerns		
		
Study	Patient selection	Index test	Reference standard	Flow and timing	Patient selection	Index test	Reference standard
Abbasi 2013^8^	U	L	L	L	L	L	L
Akyol 2016^9^	U	L	L	L	L	U	L
Lahham 2016^10^	U	L	L	L	L	L	L
Ahmadi 2016^11^	U	L	U	L	L	L	U

*QUADAS-2.* Quality Assessment of Diagnostic Accuracy Studies; *L*, low risk of bias; *U*, unclear risk of bias; *H*, high risk of bias.
