# Supplementary material for: Diagnostic Accuracy of Ultrasound for Identifying Shoulder Dislocations and Reductions: A Systematic Review of the Literature
Source: West J Emerg Med. 2017 Jul 10;18(5):937–42. doi: 10.5811/westjem.2017.5.34432 (PMC5576631; doi:10.5811/westjem.2017.5.34432)
Supplement: Supplementary file 1 [file wjem-18-937-s001.docx]

**Appendix. PubMed Search Strategy**

Limitations: None

Language Restrictions: None

Publication Date: All coverage

Keywords: [1] “shoulder dislocation”, [2] “shoulder relocation”, [3] “shoulder reduction”, [4] “ultras*”

MeSH Search Terms: [5] “shoulder dislocation”, [6] “ultrasonography”

Search strategy: 1+4, 2+4, 3+4, 5+4, 1+6, 2+6, 3+6, 5+6 with exclusion of duplicates

Date of search strategy: April 3, 2017

Total Results: 154
